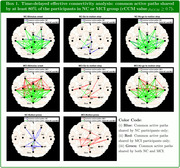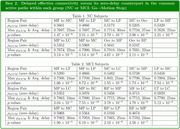# Analysis of Time‐Delayed Effective Connectivity for Normal Cognition and MCI under Motion Detection Tasks: an EEG Study

**DOI:** 10.1002/alz.088550

**Published:** 2025-01-09

**Authors:** Boxin Sun, Jinxian Deng, Alina Brighty Renli, Voyko Kavcic, Jian Ren, Bruno Giordani, Rong Zhang, Tongtong Li

**Affiliations:** ^1^ Michigan State University, East Lansing, MI USA; ^2^ Department of Neuroscience, Michigan State University, East Lansing, MI USA; ^3^ Wayne State University, Detroit, MI USA; ^4^ International Institute of Applied Gerontology, Ljubljana Slovenia; ^5^ Michigan Alzheimer's Disease Research Center, Ann Arbor, MI USA; ^6^ University of Michigan Medical School, Ann Arbor, MI USA; ^7^ UTSW Medical Center/IEEM, Dallas, TX USA; ^8^ Institute for Exercise and Environmental Medicine, Texas Health Presbyterian Hospital, Dallas, TX USA

## Abstract

**Background:**

Existing work suggested that AD pathology can affect the direction and intensity of information signaling in functional brain regions. The present study evaluates the time‐delayed effective connectivity of normal controls (NC) and patients with mild cognitive impairment (MCI) under motion‐detection tasks and explores identification of possible anomalies and deviated patterns in effective connectivity associated with AD pathology.

**Method:**

Our research focuses on task‐based EEG (64‐channel), where participants were asked to perform a motion direction discrimination task. The current dataset includes 56 consensus‐diagnosed, community‐dwelling African Americans with subjective cognitive complaints (ages 60‐90 years, 28 normal controls (NC) and 28 MCI patients) recruited through the Wayne State Institute of Gerontology and Michigan Alzheimer’s Disease Research Center.

The successive motion‐task performance periods examined include: (I) stimulus onset to Go/No‐Go indication, (II) Go (or No‐Go) indication to motion‐stop, and (III) the Button‐Press period. For each period, we evaluated the time‐delayed effective connectivity across all the possible EEG region pairs using causalized convergent cross‐mapping (cCCM) of the current source density. For each region pair, the delay (within 50ms) that results in the largest cCCM value (which reflects the intensity of the information flow) is taken as the dominant delay. Within the NC/MCI group, the directed signaling path (with cCCM value > 0.7) that appears in more than 80% of the participants is defined as a common active path of the group.

**Result:**

Our analysis shows that: (i) Delayed effective connectivity is noticeably stronger than its zero‐delay counterpart, indicating the existence of non‐zero transmission delays in inter‐region information transfer; (ii) NC and MCI may share common active paths, but each group has their own, unique common paths; (iii) Both NC and MCI groups have fewer common active paths in the Go and Button‐Press periods than during the stimulus onset and No‐Go periods, which may imply that brain effective connectivity is more focused during task execution.

**Conclusion:**

Time‐delayed effective connectivity analysis may enable identification of possible signaling pathways in the brain and of dissimilarities in pathway usage between NC and MCI associated with AD pathology.

**Funding**: NSF‐2032709/Li; NIH‐1R21AG046637‐01A1/Kavcic; NIH‐1R01AG054484‐01A1/Kavcic; NIH‐P30AG072931/Paulson; NIH‐P30AG024824/Yung.